# Cholecalciferol combined with *Satureja rechingeri* essential oils improves growth performance and immune response of broiler chickens

**DOI:** 10.1002/vms3.1587

**Published:** 2024-08-13

**Authors:** Morteza Taghizadeh, Hassan Esmaeili, Reza Vakili

**Affiliations:** ^1^ Koohrang Nature's Gold (KNG) Company Mashhad Iran; ^2^ Department of Agriculture Medicinal Plants and Drugs Research Institute Shahid Beheshti University Tehran Iran; ^3^ Animal Science Department Kashmar Branch Islamic Azad University Kashmar Iran

**Keywords:** birds, cholesterol‐lowering effects, growth, phytogenic product, vitamin D3

## Abstract

**Background:**

Vitamin D possesses an important role in the maintenance and health of broiler chickens. Herbal essential oils (EOs) have been proposed as a suitable alternative to chemical drugs in intensive production management systems for better performance of broilers with slight side effects and admirable therapeutic properties.

**Objectives:**

This experiment was conducted to investigate the effects of feeding cholecalciferol (VD) in combination of *Satureja rechingeri* EO (SREO) on growth performance, haematological indicators and immunological response of broilers.

**Methods:**

A total of 540 1‐day‐old mixed‐sex broiler chickens (Ross 308) were used in a completely randomized design with a 3 × 3 factorial arrangement of treatments. Experimental treatments included different concentrations of cholecalciferol (VD) (0, 2000 and 4000 IU/kg = 0, 0.05 and 0.1 mg/kg) and SREO (0, 200 and 400 mg/kg) on growth performance, haematological indicators and immunological responses of broiler chickens were investigated.

**Results:**

The results showed that the chicken fed diet supplemented with 0.1 mg/kg VD (VD_0.1_) in combination of 200 mg/kg SREO (SREO_200_) increased the feed intake during the overall and first 14‐day periods of the trial when compared with other dietary treatments. Interaction of VD_0.1_ × SREO_200_ led to more body weight gain (BWG) in the grower and finisher phases than all other feed treatment groups. The blood level of lymphocyte at day 42, heterophil at days 28 and 42 and heterophil/lymphocyte (H/L) ratio at 14 and 28 days of age were affected by VD_0.1_ + SREO_200_ in comparison with VD_0_ + SREO_0_ group. Feeding VD and/or SREO decreased triglyceride, cholesterol and low‐density lipoprotein concentrations at days 28 and 42 of the study, especially in VD_0.1_ + SREO_200_ treatment. Feeding VD_0.1_ + SREO_200_ also resulted in higher serum status of immunoglobulin M, lysozymes and phagocytic percentage among all treatments.

**Conclusion:**

Considering the outcomes, it is suggested that the combination of suitable concentration of VD and EO of the plant had favourable effects on the immune system and performance criteria of broiler chickens.

## Graphical abstract

The combination of cholecalciferol (VD) and *Satureja rechingeri* essential oil (SREO) enhanced growth performance, immune response and blood lipid profiles in broiler chickens. Specifically, VD at 0.1 mg/kg combined with SREO at 200 mg/kg showed increased feed intake, body weight gain and improved immune parameters compared to other dietary treatments. This study suggests that the synergistic effects of VD and SREO positively impact the health and performance of broiler chickens.

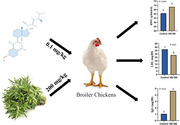

## 1 INTRODUCTION

The gradual forbiddance for the application of synthetic antibiotics in the chicken production system has led to increasing attention to alternative phytogenic feed additives to provide economic profit and eliminate the hazards of chemical antibiotics for both broilers and human consumers of broiler products (Mokhtari et al., 2018; Nasir & Grashorn, 2006; Yamashita et al., 2009). Phytobiotics represent a new generation of herbal health products or supplements, including plant‐based extracts, medicinal plants (Vakili et al., 2022), and essential oils (EOs), which are characterized by many different biological activities (Giannenas et al., 2018; Landy & Kheiri, 2023; Puvača et al., 2015). EOs are a mixture of hydrophobic volatile substances that are sometimes obtained by using pressing and distillation techniques from plant parts (leaves, flowers, herbs, seeds, fruits, bark and roots) (Brenes & Roura, 2010; Chowdhury et al., 2018; Zhai et al., 2018). Herbal EOs have been proposed as a proper alternative to synthetic drugs in intensive production management systems for better performance of broilers with slight side effects and admirable therapeutic properties (Afiouni et al., 2023; Nouri et al., 2019).

Several experiments have mentioned that the positive effect of EOs combined in poultry feed can be attributed to their antioxidant, antimicrobial, growth promoters and digestive stimulants (Platel & Srinivasan, 2004), anti‐inflammatory, antioxygenic agents (Juglal et al., 2002), hypocholesterolemic agents (Emadi et al., 2007), antiparasitic agents (Bero et al., 2014) as well as immunomodulatory functions (Valdivieso‐Ugarte et al., 2019). The EOs consumption in broiler nutrition affected the growth and immune system performance of chicken due to their biological activities. Because the antimicrobial activity of essential oils is well documented, feeding chickens with additives containing EOs can prevent microbial challenges for them and ensure the health of chickens. Therefore, in this condition, the chicken can receive and consume more feed, which leads to an increase in growth performance. These additives also help to digest food easier and faster by stimulating more digestive enzymes (Abdelli et al.,^2021^). Supplementing diet with antioxidants leads to an improve in bone strength and immune system (Khoshbin et al., 2023;Vakili et al., 2010).

Additionally, phytobiotics and EOs reduce blood cholesterol (CHO) levels (Mukhtar et al., 2013) and the abdominal fat percentage (Rafiee et al., 2013) in broilers by inhibiting CHO and lipid biosynthesis. The antibacterial activities of EOs have been thoroughly studied and widely experimented against Gram‐positive and Gram‐negative microorganisms. Among thousands of EOs constituents, thymol and carvacrol have antibacterial properties, anti‐inflammatory, antiviral and antioxidant activities, regulating the gut microbiota population and modulating immune responses.

Carvacrol is a monoterpene phenolic component obtained from herbal EOs. Investigations into the action mechanism of carvacrol demonstrated that its activity against Gram‐negative bacteria is based on the depolarization of the cytoplasmic membranes (Siroli et al., 2018; Xu et al., 2008). Furthermore, it seems that carvacrol affects ATP synthesis and the reduction of energy‐dependent cellular processes (Nostro & Papalia, 2012).

Savory (*Satureja*) consists of 17 species of annual and perennial herbaceous in Iran (Hadian et al., 2014). Different species of the genus *Satureja* in terms of the amount of EO and type of its constituent compounds show many variations. The major chemical components include carvacrol, thymol, γ‐terpinene and *p*‐cymene. *Satureja rechingeri*, with a main chemical component of carvacrol, is one of the endemic species in Iran, and its therapeutic properties can mainly be attributed to carvacrol and thymol as biologically active compounds (Esmaeili et al., 2019).

The broiler diets are formulated to provide all of the birds’ nutrient requirements under optimum growth conditions. The essential nutrients include vitamins A, E, C, D, K and B (Okafor et al., 2018). Vitamin D plays a crucial role in the maintenance and health of broiler chickens. Research in this field shows implied that vitamin D deficiency depressed the chicken immune system and makes them more susceptible to infections and diseases (Kheiri & Landy, 2019). The important and biologically active vitamin D is cholecalciferol, or vitamin D3 synthesized by a photochemical conversion of provitamin D3 (7‐dehydrocholesterol) (Fritts & Waldroup, 2003). Vitamin D3 has a role in bone development and mobilization as well as the absorption of calcium and phosphate by the kidneys (Combs et al., 1998). Because the chickens in many commercial broiler processes are grown in the presence of limited sunlight, feed additives containing cholecalciferol (vitamin D3) can help improve the growth of chickens.

According to our knowledge, no research has studied the simultaneous use of *S. rechingeri* EO (SREO) and cholecalciferol in broiler chickens. Therefore, this study was carried out for the first time to investigate the potential usage of different concentrations of SREO and cholecalciferol on growth performance, haematological parameters and immune responses in broilers.

## 2 MATERIALS AND METHODS

### 2.1 Essential oil extraction and analysis

The dried aerial part of the *S. rechingeri* plant was finely powdered, and 30 g of it was used to extract the EO through hydro‐distillation by a Clevenger‐type apparatus for 3 h pursuant to British Pharmacopoeia method. The extracted oil was injected into a Thermoquest–Finnigan gas chromatograph (GC) connected to a DB‐5 column (60 m × 0.25 mm i.d.; film thickness 0.25 µm) and a TRACE mass. Helium served as the carrier gas with an ionization voltage of 70 eV. The ion source and interface temperatures were set at 200 and 250°C, respectively. The mass range covered from 40 to 460 amu. Identification of the EO constituents involved calculating their retention indices under temperature‐programmed conditions for *n*‐alkanes (C6–C24) and the oil on a DB‐5 column under the same chromatographic conditions. Compounds were identified by comparing their mass spectra with those in the internal reference mass spectra library (Adams and Wiley 7.0) or authentic compounds. Confirmation was achieved by comparing retention indices with authentic compounds or literature references. Quantification utilized relative area percentages obtained by flame ionization detection without correction factors. The result of GC–MS analysis is presented in Table 1.

**TABLE 1:** Chemical composition of *Satureja rechingeri* essential oil.

No.	Retention index (RI)	Composition	(%)
1	933	α‐Pinene	0.5 ± 0.12
2	947	Camphene	0.3 ± 0.08
3	981	Myrcene	1.0 ± 0.17
4	1007	Delta‐3‐carene	0.1 ± 0.12
5	1013	α‐Terpinene	0.4 ± 0.03
6	1017	*p*‐Cymene	2.1 ± 0.22
7	1026	Limonene	2.5 ± 0.31
8	1036	(*Z*)‐β‐Ocimene	1.9 ± 0.21
9	1053	γ‐Terpinene	0.8 ± 0.04
10	1056	cis‐Sabinene hydrate	0.7 ± 0.04
11	1084	*trans*‐Sabinene hydrate	0.3 ± 0.02
12	1266	Thymol	0.3 ± 0.01
13	1282	Carvacrol	89.2 ± 1.02
14	1329	Thymyl acetate	0.3 ± 0.01
15	1424	β‐Caryophyllene	0.2 ± 0.01
16	1501	β‐Bisabolene	0.4 ± 0.03
17	1542	*trans*‐ˇ‐Bisabolene	0.3 ± 0.02

### 2.2 Animals and dietary treatments

The experimental study was approved by Ferdowsi University of Mashhad (IR.UM.REC.1401.286.) A total of 540 1‐day‐old mixed‐sex broiler chickens (Ross 308) were purchased from a commercial hatchery and randomly placed in equal experimental groups and reared at the recommended temperature (with an initial temperature of 30°C, then gradually decreased to 20°C) and light (18 h light and 6 h of darkness) conditions with free access to water and feed. Their maintenance and raising conditions were checked every day. This experiment was performed with 9 dietary treatments with 6 replicates (10 broilers per pen) per treatment in a completely randomized design at a research centre. The dietary treatments included three concentrations of both cholecalciferol (VD; Sigma‐Aldrich, C9756) (0, 2000, 4000 IU/kg = 0, 0.05 and 0.1 mg/kg) and *S. rechingeri* EO (MPDR institute, Tehran, Iran) (EO) (0, 200 and 400 mg/kg) during the feeding period from 1 to 42 days. The starter, grower and finisher mixtures were used for feeding the chickens in the first 14, 15–28 and 29–42 days, respectively (Table 2).

**TABLE 2:** Feed ingredients and composition of basal diet in starter, grower and finisher phases.

Feed ingredients (%)	Starter phase (days 1–14)	Grower phase (days 15–28)	Finisher phase (days 29–42)
Corn	50.17	53.10	58.30
Soybean meal	41.30	38.00	33.16
Vegtable oil	5.20	5.80	5.80
Oyster shell powder	1.10	1.04	0.85
Dicalcium phosphate	1.00	0.90	0.80
Salt	0.40	0.40	0.40
dl‐Methionine	0.30	0.28	0.20
l‐lysin HCl	0.20	0.18	0.20
l‐threonine	0.08	0.05	0.04
Vitamin and mineral premixes^a^	0.25	0.25	0.25
Calculated chemical composition			
Metabolizable energy (kcal/kg)	3020	3085	3210
Crude protein (%)	24	22.0	19.0
Methionine (%)	0.810	0.713	0.622
Methionine + cysteine (%)	1.10	0.95	0.90
Lysine (%)	1.50	1.30	1.10
Threonine (%)	0.75	0.65	0.59
Calcium (%)	0.92	0.85	0.80
Available phosphorous (%)	0.50	0.455	0.40
Sodium (%)	0.18	0.15	0.15

^a^ Vitamin premix is supplied as follows: (mg/kg diet): retinol (4.10), vitamin D (0.060), alpha tpcopherol acetat (20), pyridoxine (1), thiamine (2), niacin (30), biotin (0.1), riboflavin (5.5), choline (200), pantothenic acid (9) and folacin (0.5). Mineral premix is supplied as follows (mg/kg diet): zinc (70), iron (20), copper (15), manganese (90), iodine (2) and selenium (0.3).

### 2.3 Growth performance

For each feeding stage, the body weight gain (BWG), feed intake (FI) and feed conversion ratio (FCR) were recorded. At the end of each period (starter, grower, and finisher), the mean BWG (g/bird/day) was determined and expressed as follows: BWG = BW_f_ – BW_i_, where BW_f_ denotes the body weight at the end of the period and BW_i_ denotes the body weight of the beginning of the period. The difference between the supplied feed and uneaten feed was also used to record the (FI: g/bird/d) in every period during the rearing process. At the end of the experiment, the overall BWG and FI were computed (Al‐Dawood & Al‐Atiyat, 2022). FCR is defined as the feed consumed value (g) per BWG (g) according to the following formula: FCR = FI/BWG (Abd El‐Latif et al., 2013). The weight of the chickens and the amount of feed consumed were measured daily.

### 2.4 Serum biochemical parameters

Blood samples of four randomly selected chickens per pen were prepared using a sterile syringe for each treatment and at each stage. For haematological assays, 2 mL of sample was poured into an anticoagulant (EDTA Sigma‐Aldrich, E6758) containing tube and transferred to the laboratory. Serum samples were also obtained via centrifugation at 1500 *g* for 15 min and were stored at −20°C until analysis. Biochemical features, such as total protein (TP), triglycerides (TG), CHO, high‐density lipoprotein cholesterol (HDL), low‐density lipoprotein cholesterol (LDL), glucose (GLU), calcium (Ca) and phosphorus (P), were measured by using analytical kits (Pars Azmoon Company) based on the manufacturer's guideline according to Nahavandinejad et al. (2014). Haematological features, including red blood cells (RBC), white blood cells (WBCs), lymphocyte, heterophil, monocytes, basophil and heterophil/lymphocyte ratio, were evaluated on the basis of enzymatic method by Auto‐analyzer Bio‐Systems (Auto‐analyzer, BS‐200, MINDRAY chemistry analyzer, Germany, 2009) that previously reported by Attia et al. (2017).

### 2.5 Immune responses

The phagocytic percentage was achieved by the method previously described by Bos and de Souza (2000). An agarose gel cell‐lysis test was employed to detect lysozyme activity. The amount of immunoglobulin M (IgM) and G (IgM) in the chicken's serum was measured according to an enzyme‐linked immunosorbent assay (ELISA) kit (CK‐E20295: Hangzhou Eastbiopharm Co., Ltd.). Determinations of blood biochemical parameters and variables were performed according to the manufacturer's instructions.

### 2.6 Statistical analysis

The SAS software (version 9.4) was applied to analyse the interaction effects of VD and EO. The one‐way analysis of variance and the least squares mean procedure set for Tukey's test (*p *< 0.05, and 0.10 ≤ *p *> 0.05 as tendency) were used to determine the significance of the effect of treatments and compare their means, respectively.

## 3 RESULTS

### 3.1 Growth performance

Table 3 provides information on the impact of the VD and SREO on the growth performance of broiler chickens. Except for the first 14 days period of the trial, in which the main effect of VD was not significant (*p* > 0.10), feeding VD and/or SREO affected FI throughout the study. Feeding 0.1 mg/kg VD along with 200 mg/kg SREO increased FI during overall (*p* = 0.0003) and first 14‐day periods (*p* = 0.0007) of the trial when compared with other dietary treatments as well as higher FI in the second and third 14‐day periods of the experiment was obtained compared with VD_0_ + SREO_0_ (*p* = 0.015 or 0.039, respectively). As compared with VD_0_ birds, chicken fed with VD_0.1_ alone recorded greater FI during the second (111.6 vs. 108.9 ± 0.31 g/bird/day; *p *= 0.036), third (176.7 vs. 174.4 ± 0.23 g/bird/day; *p* = 0.050) 14‐day periods and also overall (112.57 vs. 110.2 ± 0.17 g/bird/day; *p* = 0.002). Similarly, the addition of SREO_200_ alone resulted in consuming more feed during the first (57.9 vs. 55.3 ± 0.16 g/bird/day; *p* = 0.005), second (111.6 vs. 109.0 ± 0.31 g/bird/day; *p* = 0.036), third (176.6 vs. 174.6 ± 0.23 g/bird/day; *p* = 0.049) 14‐day and overall (112.6 vs. 110.2 ± 0.17 g/bird/day; *p* = 0.001) periods than those of SREO_0_ treatment. Supplementation of VD and/or SREO had a significant effect on BWG throughout the experiment period (*p* < 0.05). Interaction of VD_0.1_ × SREO_200_ led to more BWG in the grower and finisher phases and overall than all other groups (*p* < 0.001); however, BWG during the starter phase was higher in VD_0.1_ + SREO_200_ group compared with VD_0.1_ + SREO_400_, VD_0.1_ + SREO_0_, VD_0.05_ + SREO_400_ and VD_0_ + SREO_0_ treatments (*p* < 0.05). The main effect of VD was significant for BWG (*p* < 0.05) where it was improved in broilers that received VD_0.1_ alone during the first (36.7 vs. 34.7 ± 0.61 g/bird/day; *p* = 0.046), second (63.1 vs. 60.8 ± 0.60 g/bird/day; *p* = 0.023), third (94.5 vs. 92.3 ± 0.59 g/bird/day; *p* = 0.028) 14‐day and overall (64.7 vs. 62.6 ± 0.59 g/bird/day; *p *= 0.011) periods of experiment in comparison with VD‐nontreated counterparts. Although the main effect of SREO and VD × SREO interaction was significant for FCR in the finisher phase (*p* < 0.05), no difference was found among dietary groups during the whole experimental period (*p* > 0.05). However, FCR was improved because of offering VD_0.1_ along with SREO_200_ than VD_0_ + SREO_0_ during the finisher (*p* = 0.012). Additionally, as compared with SREO_0_ birds, chickens fed with SREO_200_ only indicated better FCR in the third 14 days period (1.86 vs. 1.92 ± 0.009; *p* = 0.005) and a tendency in the second 14 days period (1.76 vs. 1.80 ± 0.012; *p* = 0.052) of the trial. Moreover, the same pattern was observed by main effect of SREO in which BWG was increased by feeding 200 mg/kg SREO only in starter (36.7 vs. 34.6 ± 0.61 g/bird/day; *p* = 0.041), grower (63.4 vs. 60.0 ± 0.60 g/bird/day; *p* = 0.002), finisher (95.0 vs. 92.0 g/bird/day; *p* = 0.0009) phases and overall periods (65.0 vs. 62.4 ± 0.59 g/bird/day; *p* = 0.001) when compared with SREO‐nontreated birds.

**TABLE 3:** Effect of cholecalciferol (Vitamin D_3_; VD) and *Satureja rechingeri* essential oil (SREO) on growth performance of broiler chickens (Ross 308).

	VD_0_			VD_0.05_			VD_0.1_				*p*‐value		
Item	SREO_0_	SREO_200_	SREO_400_	SREO_0_	SREO_200_	SREO_400_	SREO_0_	SREO_200_	SREO_400_	SEM	VD	SREO	VD × SREO
**FI (g/bird/day)**													
Starter (days 1–14)	53.4^b^	56.4^b^	56.5^b^	54.1^b^	54.7^b^	57.9^b^	58.6^b^	60.1^a^	54.8^b^	0.29	0.14	0.0007	0.004
Grower (days 15–28)	107.1^b^	110.0^b^	110.5^ab^	108.8^b^	110.3^ab^	109.9^ab^	110.9^ab^	114.7^a^	109.1^b^	0.53	0.040	0.027	0.015
Finisher (days 29–42)	173.3^b^	175.9^ab^	175.7^b^	174.4^b^	175.4^ab^	175.6^ab^	176.1^ab^	179.0^a^	175.3^b^	0.40	0.046	0.047	0.039
Overall (days 1–42)	108.9^b^	109.9^b^	109.6^b^	109.3^b^	112.9^a^	112.6^a^	110.1^b^	113.1^a^	110.0^b^	0.29	0.008	0.0005	0.0003
**BWG (g/bird/day)**													
Starter (days 1–14)	32.6^b^	35.5^ab^	35.8^ab^	35.4^ab^	35.1^ab^	34.3^b^	36.0^b^	39.6^a^	34.5^b^	1.07	0.045	0.031	0.023
Grower (days 15–28)	58.9^b^	61.8^b^	62.1^b^	61.5^b^	62.0^b^	62.1^b^	61.3^b^	67.1^a^	61.0^b^	1.03	0.034	0.002	0.006
Finisher (days 29–42)	90.3^b^	93.1^b^	93.5^b^	92.8^b^	93.3^b^	93.4^b^	92.7^b^	98.4^a^	92.3^b^	1.02	0.036	0.001	0.004
Overall (days 1–42)	60.6^c^	63.4^b^	63.8^b^	63.2^b^	63.5^b^	63.3^b^	63.3^b^	68.3^a^	62.6^bc^	1.03	0.036	0.005	0.009
**FCR**													
Starter (days 1–14)	1.64	1.59	1.58	1.53	1.56	1.69	1.63	1.52	1.59	0.045	0.84	0.22	0.11
Grower (days 15–28)	1.82	1.78	1.78	1.77	1.78	1.77	1.81	1.71	1.79	0.022	0.37	0.063	0.11
Finisher (days 29–42)	1.92^a^	1.89^ab^	1.88^ab^	1.88^ab^	1.88^ab^	1.88^ab^	1.90^a^	1.82^b^	1.90^a^	0.015	0.26	0.007	0.012
Overall (days 1–42)	1.78	1.73	1.71	1.73	1.77	1.78	1.74	1.65	1.75	0.067	0.61	0.12	0.16

Abbreviations: BWG, body weight gain; FCR, feed conversion ratio; FI, feed intake.

^a–c^Means with different superscripts within a row are different at *p* < 0.05.

### 3.2 Blood haematological features

Except for day 28 of the trial, a tendency for heterophil/lymphocyte (H/L) ratio of feeding VD_0.1_ alone was observed compared with VD‐nontreated birds (0.53 vs. 0.55 ± 0.004; *p* = 0.062), and the main effect of VD was not significant for haematological features of blood cells and H/L ratio in any time points (*p* > 0.10; Table 4). The interaction effect of VD and SREO on a total number of RBC and WBC, as well as a percentage of monocyte and basophil, was not significant at different time points (*p* > 0.10), whereas the blood level of lymphocyte at day 42 (*p* = 0.006), heterophil at days 28 and 42 (*p* = 0.091 or 0.017, respectively) and H/L ratio at days 14 and 28 (*p* = 0.045 or 0.030, respectively) of trial were affected by VD_0.1_ + SREO_200_ in comparison with VD_0_ + SREO_0_ group. Although differences in a percentage of heterophil, monocyte and basophil did not reach significance throughout the experiment period, broilers fed SREO_200_ alone showed an increase for RBC on days 28 (2.51 vs. 2.42 ± 0.022 × 10^6^/µL; *p* = 0.009) and 42 (2.73 vs. 2.65 ± 0.024 × 10^6^/µL; *p* = 0.003), WBC on day 28 (29.51 vs. 29.40 ± 0.024 × 10^3^/µL; *p* = 0.003), lymphocyte on days 14 (58.84 vs. 58.28 ± 0.14%; *p* = 0.021), 28 (60.00 vs. 59.35 ± 0.18%; *p* = 0.038) and 42 (60.00 vs. 59.36 ± 0.18%; *p* = 0.050) as well as a tendency for H/L ratio on days 14 (0.53 vs. 0.54 ± 0.003; *p* = 0.10) and 28 (0.53 vs. 0.55 ± 0.003; *p* = 0.10) of experiment relative to SREO‐nontreated chickens.

**TABLE 4:** Effect of different levels of cholecalciferol (Vitamin D_3_; VD) and *Satureja rechingeri* essential oil (SREO) on blood haematological features of broiler chickens (Ross 308).

	VD_0_			VD_0.05_			VD_0.1_				*p*‐value		
Item	SREO_0_	SREO_200_	SREO_400_	SREO_0_	SREO_200_	SREO_400_	SREO_0_	SREO_200_	SREO_400_	SEM	VD	SREO	VD × SREO
**RBC (×10^6^/µL)**													
Day 14	2.18	2.23	2.27	2.24	2.28	2.21	2.24	2.28	2.21	0.03	0.87	0.28	0.21
Day 28	2.38	2.47	2.51	2.44	2.55	2.45	2.44	2.52	2.41	0.03	0.68	0.012	0.19
Day 42	2.59	2.69	2.68	2.64	2.77	2.66	2.64	2.74	2.62	0.04	0.53	0.003	0.60
**WBC (×10^3^/µL)**													
Day 14	24.30	24.35	24.40	24.18	24.53	24.34	24.36	24.41	24.29	0.08	0.99	0.13	0.28
Day 28	26.25	26.56	26.59	26.40	26.72	26.53	26.56	26.60	26.50	0.11	0.61	0.16	0.42
Day 42	29.36	29.47	29.46	29.42	29.55	29.44	29.42	29.52	29.40	0.04	0.53	0.003	0.61
**Lymphocyte (%)**													
Day 14	58.30	58.98	59.00	58.18	58.52	58.33	58.36	59.03	59.29	0.25	0.14	0.028	0.48
Day 28	58.94	60.01	59.73	59.30	59.72	59.62	59.81	60.27	59.41	0.32	0.47	0.047	0.39
Day 42	58.12^b^	59.93^ab^	59.92^ab^	59.46^ab^	59.98^ab^	59.90^ab^	58.91^ab^	60.51^a^	59.86^ab^	0.33	0.30	0.032	0.006
**Heterophil (%)**													
Day 14	32.29	31.98	30.94	31.30	31.52	31.33	31.36	30.89	31.29	0.37	0.18	0.31	0.20
Day 28	33.44	32.96	31.88	32.24	32.47	32.43	32.34	31.74	32.39	0.39	0.17	0.38	0.091
Day 42	33.78^a^	33.30^ab^	31.80^b^	32.58^ab^	32.81^ab^	32.77^ab^	32.68^ab^	31.71^b^	32.73^ab^	0.43	0.24	0.24	0.017
**Monocyte (%)**													
Day 14	4.60	4.65	4.69	4.48	4.82	4.63	4.66	4.70	4.59	0.08	0.99	0.13	0.28
Day 28	4.63	4.74	4.78	4.57	4.91	4.72	4.75	4.75	4.68	0.09	0.97	0.13	0.29
Day 42	4.06	4.17	4.16	4.12	4.17	4.14	4.12	4.18	4.10	0.05	0.96	0.26	0.85
**Basophil (%)**													
Da 14	2.28	2.84	2.85	3.52	2.61	3.19	3.10	2.87	3.31	0.52	0.49	0.72	0.73
Day 28	2.77	2.07	3.40	3.68	2.68	3.00	2.88	3.03	3.30	0.58	0.69	0.36	0.67
Day 42	2.96	3.18	2.66	3.72	2.93	3.07	3.54	2.90	3.19	0.41	0.59	0.35	0.72
**H/L ratio**													
Day 14	0.553^a^	0.541^ab^	0.525^b^	0.538^ab^	0.538^ab^	0.537^ab^	0.537^ab^	0.523^b^	0.536^ab^	0.006	0.29	0.091	0.045
Day 28	0.567^a^	0.548^ab^	0.534^b^	0.543^ab^	0.543^ab^	0.544^ab^	0.541^ab^	0.527^b^	0.545^ab^	0.006	0.078	0.097	0.030
Day 42	0.563	0.555	0.547	0.548	0.547	0.547	0.558	0.528	0.546	0.007	0.22	0.10	0.29

Abbreviations: H/L, heterophil/lymphocyte; RBC, red blood cell; WBC, white blood cel.

^a,b^Means with different superscripts within a row are different at *p* < 0.05.

### 3.3 Blood biochemical attributes

Dietary treatments did not affect the serum biochemical attributes on day 14 of the trial (*p* > 0.10; Table 5). Likewise, serum TP and GLU levels of all chickens remained unchanged throughout the study (*p* > 0.10). Feeding VD_0.1_ along with SREO_200_ led to a lower CHO level at day 14 of the trial compared with other experimental groups, in addition to a notable decrease on day 42 of the trial than the VD_0_ + SREO_0_ group (*p *< 0.05). At days 28 and 42 of the trial, VD_0.1_‐treated birds had a lower concentration of serum CHO than VD_0_‐treated ones (118.6 vs. 120.2 ± 0.39 mg/dL; *p* = 0.009 and 119.5 vs. 121.1 ± 0.37 mg/dL; *p* = 0.013, respectively). Similarly, SREO_200_‐fed broilers indicated a lower CHO level compared with SREO_0_‐fed broilers in both days 28 (118.5 vs. 120.1 ± 0.39 mg/dL; *p* = 0.024) and 42 (119.5 vs. 121.0 ± 0.37 mg/dL; *p* = 0.013) of trial. On day 28 of the trial that was not significant VD_0.05_ + SREO_200_ birds, VD_0.1_ + SREO_200_ broilers had markedly decreased levels of serum TG compared to other dietary treatments (*p* < 0.05). However, VD_0.1_ + SREO_200_ chickens compared to VD_0_ + SREO_0_ showed a lower serum TG at day 42 of the experiment (*p* < 0.05). On both days 28 and 42 of the trial, serum TG level was influenced by the main effect of VD or SREO, in which at these times feeding VD_0.1_ alone than VD‐nontreated chickens (73.66 vs. 75.4 ± 0.39 mg/dL; *p* = 0.006 for day 28 and 74.5 vs. 76.0 ± 0.37 mg/dL; *p* = 0.013 for day 42) or feeding SREO_200_ alone than SREO‐nontreated chickens (73.7 vs. 75.2 ± 0.39 mg/dL; *p* = 0.035 for day 28 and 74.5 vs. 76.0 ± 0.37 mg/dL; *p* = 0.015 for day 42) let to decreased concentration of serum TG. Although no interaction was observed for serum HDL concentration at days 14 and 42 of the trial (*p* > 0.10), it was numerically affected by the interaction of VD and SREO on day 28 of the trial (*p* = 0.074). In addition, the supplementation of VD_0.1_ only relative to the VD_0_ group was elevated significantly serum HDL on day 28 of the trial (72.44 vs. 70.6 ± 0.59 mg/dL; *p* = 0.049) as well as numerically on day 42 of the trial (73.4 vs. 72.1 ± 0.51 mg/dL; *p *= 0.092), whereas offering SREO_200_ alone compared with SREO‐nontreated birds was increased concentration of serum HDL at days 28 and 42 of the trial (72.5 vs. 70.5 ± 0.59 mg/dL; *p* = 0.038 and 73.5 vs. 71.2 ± 0.51 mg/dL; *p* = 0.045, respectively). Moreover, in the same pattern with CHO and TG, feeding VD and/or SREO decreased LDL concentration at days 28 and 42 of the study (*p* < 0.01). The main effect of SREO and the interaction of VD and SREO did not affect serum concentration of Ca and P at different time points (*p* > 0.05). However, chicken fed VD_0.1_ and VD_0.05_ alone had higher serum Ca and P than VD_0_ birds on days 28 (13.13, 12.76 vs. 10.89 ± 0.52 mg/dL; *p* = 0.007 and 6.84, 6.76 vs. 5.64 ± 0.28 mg/dL; *p* = 0.003, respectively) and 42 (13.66, 13.29 vs. 11.43 ± 0.54; *p* = 0.004 and 7.21, 7.13 vs. 6.01 ± 0.29; *p* = 0.005, respectively) of experiment.

**TABLE 5:** Effect of different levels of cholecalciferol (Vitamin D_3_; VD) and *Satureja rechingeri* essential oil (SREO) on blood biochemical attributes in broiler chickens (Ross 308).

	VD_0_			VD_0.05_			VD_0.1_				*p*‐Value		
Item	SREO_0_	SREO_200_	SREO_400_	SREO_0_	SREO_200_	SREO_400_	SREO_0_	SREO_200_	SREO_400_	SEM	VD	SREO	VD × SREO
**CHO (mg/dL)**													
Day 14	121.01	120.55	119.95	119.61	119.57	121.55	120.82	119.24	120.32	0.80	0.84	0.41	0.28
Day 28	121.01^a^	119.97^a^	119.82^a^	119.61^a^	119.45^a^	121.13^a^	119.57^a^	116.32^b^	119.20^a^	0.68	0.006	0.005	0.029
Day 42	122.01^a^	120.76^a^	120.41^ab^	120.41^ab^	120.03^ab^	121.71^a^	120.49^ab^	117.70^b^	120.49^ab^	0.64	0.012	0.008	0.044
**TG (mg/dL)**													
Day 14	76.00	75.55	75.36	75.44	74.57	76.96	76.23	75.49	75.73	0.91	0.96	0.50	0.61
day 28	76.01^a^	75.38^a^	74.82^a^	75.03^a^	74.45^ab^	76.13^a^	74.57^a^	71.53^b^	74.90^a^	0.69	0.004	0.013	0.042
Day 42	77.00^a^	75.76^a^	75.40^ab^	75.40^ab^	75.03^ab^	76.71^a^	75.48^ab^	72.69^b^	75.48^ab^	0.64	0.013	0.007	0.045
**HDL (mg/dL)**													
Day 14	71.22	71.27	71.31	71.36	71.32	71.25	71.28	71.32	71.25	0.06	0.69	0.83	0.59
Day 28	67.63	71.89	72.48	72.50	72.00	72.81	71.40	73.79	72.23	1.02	0.049	0.021	0.074
Day 42	69.88	72.89	73.48	73.50	73.00	73.81	72.40	74.79	73.23	0.89	0.096	0.040	0.14
**LDL (mg/dL)**													
Day 14	34.57	34.16	33.55	33.15	33.33	34.89	34.28	32.81	33.91	0.68	0.73	0.42	0.21
Day 28	38.16^a^	32.99^ab^	32.37^b^	32.10^b^	32.55^b^	33.08^ab^	33.25^ab^	28.21^b^	32.68^b^	1.22	0.007	0.005	0.010
Day 42	36.71^a^	32.70^abc^	31.84^bc^	31.81^bc^	32.02^bc^	32.55^abc^	32.98^ab^	28.35^c^	32.15^bc^	1.01	0.008	0.003	0.011
**TP (mg/dL)**													
Day 14	4.52	4.69	4.52	4.59	4.63	4.34	4.58	4.56	4.74	0.12	0.59	0.63	0.37
Day 28	4.60	4.73	4.56	4.64	4.67	4.39	4.62	4.58	4.74	0.13	0.73	0.67	0.46
Day 42	4.56	4.73	4.52	4.64	4.67	4.39	4.58	4.62	4.83	0.13	0.60	0.62	0.26
**GLU (mg/dL)**													
Day 14	166.39	166.85	166.39	167.03	166.43	167.03	166.45	166.43	166.45	0.82	0.84	0.99	0.96
Day 28	166.14	167.02	166.97	167.20	166.59	167.19	167.45	167.01	166.58	0.97	0.91	0.99	0.88
Day 42	166.72	167.17	166.71	166.94	166.75	166.93	166.36	166.75	166.36	0.96	0.85	0.94	0.99
**Ca (mg/dL)**													
Day 14	11.06	11.68	11.07	12.94	11.26	11.94	11.34	11.50	12.59	0.80	0.48	0.78	0.83
Day 28	8.81	11.28	12.56	13.44	12.93	13.02	12.84	12.17	13.26	0.90	0.007	0.23	0.15
Day 42	9.38	11.81	13.10	13.97	13.46	13.56	13.80	12.68	13.38	0.94	0.004	0.44	0.13
**P (mg/dL)**													
Day 14	5.62	5.94	5.63	6.07	6.23	6.07	5.77	5.84	6.36	0.40	0.47	0.80	0.85
Day 28	4.61	5.84	6.48	6.92	6.66	6.71	6.63	7.07	6.83	0.48	0.003	0.26	0.23
Day 42	4.99	6.20	6.84	7.28	7.04	7.07	7.21	7.43	6.99	0.50	0.005	0.46	0.20

Abbreviations: Ca, calcium; CHO, total cholesterol; GLU, glucose; HDL, high‐density lipoprotein; LDL, low‐density lipoprotein; P, phosphorus; TG, triglyceride; TP, total protein.

^a‐c^Means with different superscripts within a row are different at *p* < 0.05.

### 3.4 Serum immune parameters

The results of the interaction of VD and SREO on the serum immune factors are shown in Table 6. Feeding VD_0.1_ + SREO_200_ resulted in higher serum status of IgM (*p* = 0.036), lysozymes (*p* = 0.001) and phagocytic percentage (*p* = 0001) compared with other experimental groups as well as an increased level of serum IgG in VD_0.1_ + SREO_200_ group relative to VD_0.1_ + SREO_400_, VD_0.05_ + SREO_400_, VD_0.05_ + SREO_0_ and VD_0_ + SREO_0_ groups (*p* < 0.05). Furthermore, the main effect of VD or SREO was significant for tested immune responses, where VD_0.1_ broilers had higher serum levels of IgM (15.80 vs. 14.55 ± 0.35; *p* = 0.033), IgG (3.24 vs. 2.02 ± 0.30 mg/dL; *p* = 0.015), lysozymes activity (3.22 vs. 2.03 ± 0.28 µ/mL; *p* = 0.009) and phagocytic percentage (66.22 vs. 65.00 ± 0.28%; *p* = 0.007) compared with VD_0_ ones. Likewise, SRE_200_ chickens had higher serum levels of IgM (15.82 vs. 14.59 ± 0.35; *p* = 0.038), IgG (3.15 vs. 2.04 ± 0.30 mg/dL; *p* = 0.031), lysozymes activity (3.28 vs. 1.97 ± 0.28 µ/mL; *p* = 0.003) and phagocytic percentage (66.28 vs. 64.93 ± 0.28%; *p* = 0.002) compared with SREO_0_ birds.

**TABLE 6:** Effect of different levels of cholecalciferol (Vitamin D_3_; VD) and *Satureja rechingeri* essential oil (SREO) on serum immune parameters in broiler chickens (Ross 308).

	VD_0_			VD_0.05_			VD_0.1_				*p*‐Value		
Item	SREO_0_	SREO_200_	SREO_400_	SREO_0_	SREO_200_	SREO_400_	SREO_0_	SREO_200_	SREO_400_	SEM	VD	SREO	VD × SREO
**IgM (mg/dL)**	13.10^b^	15.41^ab^	15.15^ab^	15.17^ab^	14.90^ab^	14.51^ab^	15.52^ab^	17.15^a^	14.74^ab^	0.61	0.034	0.032	0.036
**IgG (mg/dL)**	1.04^b^	2.41^ab^	2.60^ab^	2.17^b^	2.39^ab^	2.28^b^	2.92^ab^	4.65^a^	2.13^b^	0.53	0.014	0.033	0.044
**Lysozymes (µ/mL)**	1.02^b^	2.46^b^	2.61^b^	2.58^b^	2.28^b^	2.36^b^	2.31^b^	5.12^a^	2.24^b^	0.49	0.011	0.004	0.001
**Phagocytic (%)**	63.90^b^	65.46^b^	65.61^b^	65.58^b^	65.28^b^	65.36^b^	65.31^b^	68.12^a^	65.24^b^	0.50	0.009	0.003	0.001

^a,b^Means with different superscripts within a row are different at *p *< 0.05.

## 4 DISCUSSION

Today, the use of plant EOs is highly regarded in the poultry industry as a growth enhancer due to their safety, availability and naturalness (Rahimi et al., 2021). The positive effects of EOs and cholecalciferol in the poultry industry have been mentioned separately in many studies by researchers. This study identified the beneficial dose‐dependent interaction of VD and SREO on broiler chicken immune response, serum biochemical profile and growth performance. The combined consumption of VD_0.1_ + EO_200_ showed a stimulating effect on chicken growth performance. Although some reports have shown beneficial impacts of VD supplementation on BW and FI of birds (Edwards et al., 2002; Fritts & Waldroup, 2005; Han et al., 2016; Snow et al., 2004), other studies showed no effect of vitamin D on muscle development and growth performance (Adedeji et al., 2018; Colet et al., 2015). Santiago et al. (2016) reported that dietary supplementation with VD boosted the BW and feed efficiency, whereas it reduced the mortality rate. On the other hand, although some articles claim that EOs have no influence on the growth parameters of chickens, the promoting effect of plant EOs on the growth performance of broiler chickens has been previously reported. In this regards, different concentrations of EOs from 25 mg/kg (Attia et al., 2019) to 1200 mg/kg (Roofchaee et al., 2011) were employed to study their effect on the performance of broiler chickens. In a study conducted by Hashemipour et al. (2013), the maximum feed efficiency and BW were achieved in a treatment containing 200 mg/kg thymol + carvacrol. The application of 60 mg/kg thyme EO increased growth and decreased FCR in quail (Denli et al., 2004). An increase in the growth of broiler chickens from 2546 to 2617 g has been observed with a consumption of 10 mg/kg thyme extract. This increase in growth reached 2882 g with the consumption of 20 mg/kg (Al‐Kassie et al., 2009). The experiment conducted by Toghyani et al. (2010) determined that the effect of thyme powder on the BW of chickens depends on the concentration, wherein the lower concentration of thyme powder was more effective in improving BW and reducing FCR. In fact, how to respond to the use of EOs in poultry production depends on the type and dose of EOs and the health status of the birds (Puvača et al., 2022). Different mechanisms have been proposed regarding the growth‐promoting effect of EOs on broiler chickens, including intensifying the secretion of digestive enzymes (Pinheiro et al., 2004; Popović et al., 2016), disinfection of feed (Giannenas et al., 2003; Zhang et al., 2015), help to improve the absorption of substances (Diaz Carrasco et al., 2019; Windisch et al., 2008), improving the mucin secretion in the intestine (Jamroz et al., 2006), antioxidant and anti‐inflammatory effects (Pirgozliev et al., 2019) and stabilization of intestinal pH (Puvača et al., 2022). EOs cause better absorption of essential nutrients by not only reducing undesirable microorganisms, but also by activating beneficial microorganisms in the digestive system of broiler chickens (Kroismayr et al., 2008). Carvacrol present in the EOs of *S. rechingeri* is well known as an antimicrobial compound that can improve digestion and absorption of nutrients by affecting microorganisms in the digestive tract of broiler chicken. It has also been found that a nutritional mixture containing carvacrol improves the absorption of amino acids (Jamroz et al., 2005). The digestive‐stimulating, antioxidant and immunomodulatory effects of carvacrol were reported in previous publications (Puvača et al., 2022). The positive effect of a mixture containing carvacrol in improving the growth performance of broiler chickens has also been previously reported (Choi et al., 2022). An 8.1% and 7.7% increment in daily gain and FCR, respectively, were observed in 17‐day‐old poults feeding with 300 mg/kg carvacrol (Jamroz et al., 2002). However, it is not completely clear that above‐mentioned mechanism is more effective in poultry production.

In this study, the treatment of VD_0.1_ + EO_200_ increased the lymphocyte percentage, whereas it decreased the level of heterophil in the finisher stage (day 42). Thereby, the H/L ratio was also decreased under VD_0.1_ + EO_200_ treatment in the starter and grower stages (days 14 and 28, respectively) compared with VD_0_ + EO_0_. The levels of RBC, WBC, basophil and monocytes were not significantly affected by dietary additives containing different mixtures of VD and EO. Silva‐Vázquez et al. (2018) reported that leukocyte and lymphocyte numbers were elevated with the administration of Mexican oregano EO. According to Attia et al. (2019) and Geravand et al. (2021) results, none of the blood haematological features of broiler chicken, such as RBC, WBC, basophil, monocytes, lymphocyte and heterophil, were affected by the EO (consisting of 4.5 g cinnamaldehyde and 13.5 g thymol per 100 g of EOs) and licorice EO, respectively. As previously reported, the administrating of thymol and carvacrol in broiler chicken did not change the number of RBC, whereas it reduced the H/L ratio (Wójcik et al., 2019). They also showed that thyme EO had the lowest H/L ratio compared with other treatments. The H/L ratio is introduced as a stress index. Therefore, in the present study, the application of VD_0.1_ + EO_200_ has provided stable conditions for chick feeding and reduced possible stresses.

In this study, the treatment of VD_0.1_ + EO_200_ showed CHO‐lowering effects in the grower and finisher stages. This treatment was also effective in reducing TG and LDL levels. In fact, all treatments, including VD and SREO, decreased the LDL level compared with the control (VD_0_ + EO_0_). The role of some EOs in lowering blood CHO has already been identified. Some reports indicate that the amount of blood CHO, TG, LDL and HDL is dependent on the dosage of plant extracts (Aghazadeh et al., 2011; Ghazalah & Ali, 2008). Ghazalah and Ali (2008) indicated that LDL level was reduced from 54 to 36.25 mg/dL by consuming rosemary 1%. In the present study, LDL altered from 36.48 mg/dL in control to 29.79 mg/dL in VD_0.1_ + EO_200_ treatment. To justify this phenomenon, it has been stated that plant EOs have a stimulating role in the expression of 3‐hydroxy‐3‐methyl glutaryl‐CoA reductase protein, which itself reduces LDL and CHO levels in the blood of birds (Jazi et al., 2018). The study of some researchers also supports the theory that plant EOs have no effect on CHO, TG and LDL concentrations, contrary to these mentioned results. For example, Toghyani et al. (2010) reported that feeding broiler chicken with thyme powder did not effect on these parameters, whereas Bolukbasi et al. (2006) observed an increase in TG, LDL and HDL concentrations in broiler chicken.

In this study, an interaction effect of VD and SREO in a specific concentration (VD_0.1_ + EO_200_) incited immunoglobulin levels and increased lysozyme activity and phagocytic percentage. Similar results regarding the impact of EOs on the immune parameters of birds have been reported previously (Abou‐Elkhair et al., 2014; Kishawy et al., 2022; Zhai et al., 2018). The results about the effect of vitamin D on the immune response of broiler chickens have emphasized its positive effect (Aslam et al., 1998; Beal et al., 2004; Gómez‐Verduzco et al., 2013; Khan et al., 2023; Takahashi et al., 2002). Carvacrol, as the main component of SREO, has been proven to have antioxidant, antibacterial and anti‐inflammatory activities, which promote serum immunity parameters resulting in digestive system health, reducing the number of unwanted bacteria and avoiding inflammation in chicken.

## 5 CONCLUSION

In this study, the beneficial effects of the combined application of VD and SREO on enhancing the performance and strengthening the immune system of broiler chickens were proved as a first report. This combined diet with a suitable concentration of both also showed favourable effects in reducing blood TGs and CHO in chickens. In conclusion, adding 200 mg/kg SREO and 0.1 mg/kg of VD caused favourable results among all treatment groups, which can be used to produce more and healthier chickens.

## AUTHOR CONTRIBUTIONS

**Morteza Taghizadeh**: Conceptualization; formal analysis; investigation; methodology; validation; writing—original draft. **Hassan Esmaeili**: Conceptualization; data curation; investigation; supervision; writing—review & editing. **Reza Vakili**: Writing—review & editing.

## CONFLICT OF INTEREST STATEMENT

The authors declare no conflicts of interest.

## FUNDING INFORMATION

The Authors received no funding for this work.

### ETHICS STATEMENT

All research reported in this research has been conducted in an ethical and responsible manner and is in full compliance with all relevant codes of experimentation and legislation.

### PEER REVIEW

The peer review history for this article is available at https://publons.com/publon/10.1002/vms3.1587.

## Data Availability

The data presented in this study are available on request from the corresponding author upon reasonable request.
